# Changes of inflammatory mediators and oxidative stress indicators in children with Henoch-Schönlein purpura and clinical effects of hemoperfusion in the treatment of severe Henoch-Schönlein purpura with gastrointestinal involvement in children

**DOI:** 10.1186/s12887-019-1802-2

**Published:** 2019-11-04

**Authors:** Ying Zhu, Yang Dong, Lin Wu, Fang Deng

**Affiliations:** 1 Department of Nephrology, Anhui Provincial Children’s Hospital, No. 39 Wangjiang East Road, Hefei, 230051 China; 2 Department of Pediatrics, The First Affiliated Hospital of Anhui Medical University, Hefei, 230022 China

**Keywords:** Henoch-Schönlein purpura, Oxidative stress, Inflammatory mediators, Hemoperfusion, Children

## Abstract

**Background:**

To explore the changes of inflammatory and oxidative stress responses in Henoch-Schönlein purpura (HSP) children, and further analyzed the therapeutic effects and mechanisms of hemoperfusion (HP) on HSP with severe gastrointestinal (GI) involvement.

**Methods:**

There were 200 children with HSP were divided into three groups according to their clinical manifestations: 60 in HSP without GI and renal involvement group, 60 in HSP with GI involvement group, and 80 in HSPN group. The HSP with GI involvement group was subdivided into conventional treatment (*n* = 30) and HP (*n* = 30) groups. Thirty children who visited the department of children healthcare for healthy physical examinations from January to December 2017 were set as healthy control group. The IL-6 and TNF-α levels were detected by chemoluminescence method. The MDA, SOD and T-AOC levels were determined by thiobarbituric acid colorimetric method, hydroxylamine method and chemical colorimetry.

**Results:**

Compared with healthy group, IL-6, TNF-α and MDA levels in HSP were increased in each group, while SOD and T-AOC were decreased (*P* = 0.000). IL-6, TNF-α and MDA levels in the HSPN group were the highest, SOD and T-AOC levels were the lowest (*P* = 0.000). Compared with those before treatment, IL-6, TNF-α and MDA levels in the conventional and HP groups were decreased and SOD and T-AOC levels were increased (*P* = 0.000). The changes in HP group were more significant than those in conventional group (*P* < 0.047). Compared with conventional group, glucocorticoid dosage and the occurrence rate of hematuria and/or proteinuria within 3 months were lower in HP group. (*P* = 0.000, 0.004).

**Conclusions:**

Inflammatory and oxidative stress may be involved in the acute phase of HSP children. The intensity of inflammatory and oxidative stress responses were related to the degree of renal involvement. HP can reduce glucocorticoid dosage and the rate of renal involvement in children with severe HSP with GI involvement. The mechanism may be related to the fact that HP can effectively remove IL-6, TNF-α, MDA in HSP children.

## Background

Henoch-Schönlein purpura (HSP) is one of the most common systemic vasculitis in childhood, which often invades the skin, gastrointestinal (GI) tract, joints, kidneys, a ccounting for an incidence of about 6–24/100,000 per year [1–3]. HSP occurs to males more than to females, and the ratio between boys and girls is approximately 1.4:1 [1]. It is commonly observed in preschool and school-age children, and its peak incidence is observed between 2 and 6 years age-group, and 90% of the patients below10 years age [1–3]. Among them, about 50 to 75% of children had GI symptoms, and may also have refractory abdominal pain, GI bleeding, and may be accompanied by complications such as intestinal obstruction, intussusception and intestinal perforation and others in some severe cases [4–6], which in turn affected the early prognosis and remains difficult to treat. About 20 to 60% of the patients have renal involvement, which is known as Henoch-Schönlein purpura nephritis (HSPN) [7–12], and the severity of renal involvement affects the long-term prognosis of children with HSP [8–12]. The manifestations of HSPN vary from asymptomatic hematuria and/or proteinuria to nephritis syndrome, nephrotic syndrome, and rapidly progressive glomerulonephritis [8–12]. Renal biopsy is recommended if proteinuria occurs in children with HSPN [7].

The exact etiology and pathogenesis of HSP have not yet been fully elucidated. The pathogenesis of HSP is often associated with abnormal expression of inflammatory cytokines [13–15], oxidative stress [16], abnormal glycosylation of IgA1 [17], and genetic factors [13, 18]. Inflammatory mediators such as interleukin-6 (IL-6) [19], tumor necrosis factor-α (TNF-α) [15] and lipid peroxidation product malondialdehyde (MDA) [16, 20] played an important role in the initiation of inflammatory responses in HSP. The therapeutic effects of simple rash and articular purpura remained relatively good [21]. Currently, the treatment of severe HSP with GI and renal involvement depends on glucocorticoids, but it still remains difficult to be effective in some refractory cases. This may be due to that the inflammatory mediators and the oxidative stress products could not be eliminated timely in HSP.

AS a new technology invented in recent years, Hemoperfusion (HP) is applied to eliminated the inflammatory mediators and circulating immune complexes in the blood of HSP patients through extracorporeal circulation [20, 22–25]. However, the mechanisms of HP in the treatment of severe HSP still remained unclear. Therefore this study has tried to explore the changes of inflammatory responses and oxidative stress in HSP children and its corelation to organ injury, and to further the study of therapeutic effects and mechanisms of HP on severe HSP with GI involvement. By comparing the levels of IL-6, TNF-α, MDA, superoxide dismutase (SOD), and total anti-oxidant capability (T-AOC) in the acute phase of HSP children with different clinical manifestations, and the correlation between IL-6, TNF-α, MDA and renal pathological grade, as well as comparison of the changes of IL-6, TNF-α, MDA, SOD and T-AOC levels before and after HP treatment in children with severe HSP with GI involvement and the clinical outcomes. And this study has summarized the effect of HP on glucocorticoid dosage and renal involvement to provide theoretical basis for clinical HP treatment of severe HSP with GI involvement.

## Methods

### Study subjects and inclusion criteria

A total of 200 HSP cases from the Department of Nephrology, Anhui Provincial Children’s Hospital from January 2016 to June 2018 were selected as study subjects. There were 125 males and 75 females, aged 3–16 years old (7.73 ± 2.77 years). Grouping: (1) These patients were divided into 3 groups according to the clinical manifestations: 60 patients in the HSP without GI and renal involvement group, 60 patients in HSP with GI involvement group, and 80 patients in the HSPN group. Thirty children who visited the department of children healthcare for healthy physical examinations from January to December 2017 were set as healthy control group. (2) The HSP with GI involvement group was divided into two subgroups according to whether HP treatment was performed: children in the conventional treatment group (30 cases) only received medical treatment and those in the HP group (30 cases) received HP treatment (once a day for 3 consecutive days) from day 2 after admission on the basis of conventional treatment. Inclusion criteria were as follows: (1) The diagnostic criteria of HSP patients are based on the EULAR/PRINTO/PRES in 2008 [26]. The diagnostic criteria of HSPN are based on ISKDC in 1974 and Nephrology group, Chinese branch of pediatrics, Chinese medical association in 2016 [7, 27]. (2) All children were first-onset cases without history of HSP and other chronic diseases. No children used glucocorticoids and antioxidants before undergoing examinations. The HSP without GI and renal involvement was manifested as simple rash and/or mild arthralgia. The HSP with GI involvement was manifested as active bleeding of digestive tract and/or persistent abdominal pain with or without intestinal complications (such as intussusception, intestinal obstruction, intestinal perforation, pancreatitis). There were moderate to high levels of proteinuria (> 25 mg/kg.d^− 1^) with/without hematuria in the HSPN group. (3) There was no occurrence of hematuria and proteinuria in the HSP without GI and renal involvement group and HSP with GI involvement group. Renal biopsy was performed for all HSPN patients. The study was approved by the Medical Research Ethics Committee of Anhui Provincial Children’s Hospital, informed consent was obtained from all guardians of individual participants included in the study.

### Therapeutic regimen

Patients in the acute phase were treated according to the treatment guidelines [1, 28]: methylprednisolone was used to inhibit the immune responses for articular and HSP with GI involvement in the acute phase. Children in the HSPN group were treated with methylprednisolone and/or immunosuppressive agents according to the disease condition, children with proteinuria were administrated with angiotensin converting enzyme inhibitor (ACEI) and angiotensin receptor blocker (ARB) drugs. Seldinger technique was applied in the HP treatment, and a single-needle double-lumen catheter was inserted into the femoral vein to establish a temporary vascular access. The JA-800A hemoperfusion machine (Zhuhai Lizhu Biomaterial Co., Ltd.) was used and the HA280 resin hemoperfusion device was selected for HP treatment. The treatment duration was 2 h/time.

### Observational indicators

Gender, age, clinical parameters were recorded for all patients. For the HSP with GI involvement, the remission of rash, abdominal pain and digestive tract bleeding were observed before treatment (d0) and on day 4 after treatment (d4) in the conventional group of children, as well as before HP treatment (d0) and on day 4 after HP treatment for 3 times (d4) in the HP group of children. The dosage of methylprednisolone, length of hospital stay, hospital costs and complications of HP as well as high dose methylprednisolone were summarized, the relapse of rash and/or abdominal pain and the appearances of hematuria and/or proteinuria were statistically analyzed. All patients were followed up for at least 3 months (at 2 weeks, 1 month, 2 months and 3 months after discharge). The follow-up was performed by return visit or telephone interview, and the discussion included information regarding control or relapse of rash and abdominal pain, and results of urine routines.

### Detection of IL-6, TNF-α, MDA, SOD and T-AOC levels

Venous blood of 3 mL × 2 samples were collected before treatment from all HSP children, on day 4 (d4) after treatment to detect HSP with GI involvement children of the conventional group and HP group, and after physical examinations for the children in the healthy control group. One blood sample was centrifuged at 4000 r/min for 5 min at 4 °C. The supernatant was stored at − 80 °C. The levels of serum IL-6 and TNF-α were detected by chemiluminescence method (the kit was purchased from Siemens). The other blood sample was centrifuged at 2000 r/min for 15 min at 4 °C. The plasma was then separated (stored at − 80 °C) to detect the MDA levels (thiobarbituric acid colorimetric method), SOD levels (hydroxylamine method) and T-AOC levels (chemical colorimetry) (the kits were purchased from Nanjing Jiancheng Institute of Bioengineering).

### Kidney biopsy

Eighty children with HSPN underwent renal histopathological examinations taking the inferior pole of the right kidney as the puncture point under the guidance of ultrasound. The kidney tissues were taken out for light microscopy, immunofluorescence and electron microscopy.

### Statistical analysis

Data was processed using SPSS 17.0 software. The measurement data was expressed as means±standard deviation (^−^*x* ± *s*). One-way analysis of variance was used for comparisons between multiple groups. The t-test was used for pair-wise comparisons between groups. The enumeration data was expressed as percentages, and the comparisons between the groups were performed by row x column table χ^2^ tests. The correlation tests between the two variables were analyzed by Pearson linear correlation analysis. *P* < 0.05 was considered to be statistically significant.

## Results

### Demographics, clinical parameters in each HSP group and healthy control group

There were no significant differences in gender, age, complement3 (C3) and fibrinogen (Fib) between the groups. Compared with the health group, the levels of C-reactive protein (CRP) and immunoglobulin A (IgA) in each HSP group were higher (*P* < 0.02, *P* = 0.000). Compared with the HSP without GI and renal involvement group, the levels of white blood cell count (WBC) were higher, while albumin (ALB) were lower in the HSP with GI and HSPN groups (*P* = 0.000, *P* < 0.001). Compared with the HSPN group, the levels of serum creatinine (Scr) were lower in HSP without GI and renal involvement and HSP with GI groups (*P* = 0.011, 0.041) (Table 1).

**Table 1:** Comparison of demographics, clinical parameters between each HSP group and healthy control group [cases(%),^−^*x ± s*]

	HSP-GI and renal involvement(*n* = 60)	HSP+ GI involvement(*n* = 60)	HSPN(*n* = 80)	Healthy group(*n* = 30)	*P* value
Age (years)	7.22 ± 2.38	7.58 ± 3.17	8.21 ± 2.28	8.31 ± 2.05	0.078
Gender(M/F)	35/25	37/23	53/27	17/13	0.727
Abdominal pain	0(0)	60(100)	48(60)	0(0)	0.000
Arthralgia	54(90)	24(40)	38(48)	0(0)	0.000
WBC(× 10^9^/L)	9.48 ± 2.96	14.71 ± 6.02	12.99 ± 6.65	8.31 ± 2.05	0.000
CRP(mg/L)	6.25 ± 7.22	11.05 ± 13.70	10.82 ± 12.33	3.13 ± 2.41	0.001
ALB(g/L)	43.46 ± 3.74	39.66 ± 6.57	37.68 ± 6.83	45.44 ± 3.27	0.000
SCr (μmol/L)	29.2 ± 6.89	29.3 ± 9.63	34.13 ± 11.27	29.14 ± 4.32	0.002
IgA(g/L)	2.08 ± 1.00	1.93 ± 0.65	2.21 ± 0.82	0.93 ± 0.14	0.000
C3(g/L)	1.05 ± 0.23	1.07 ± 0.26	1.02 ± 0.26	1.08 ± 0.18	0.664
Fib (g/L)	2.73 ± 0.60	2.86 ± 1.98	2.43 ± 0.76	2.65 ± 0.47	0.179

### Clinical parameters and biopsy results in HSPN patients

No significant difference were found in age, gender, 24 h urine protein excretion (24hUPro) and urine red blood cell counts (URBC) in HSPN group with different pathological grades (Table 2).

**Table 2:** Clinical parameters and Histopathological examination in HSPN patients [cases(%),^−^*x ± s*]

	I	IIa	IIb	IIIa	IIIb	*P* value
Cases(n)	1(1)	21(26)	6(8)	49(61)	3(4)	
Age(years)	13	8.10 ± 2.32	7.67 ± 2.73	8.35 ± 2.24	7.67 ± 3.06	0.305
Gender(M/F)	0/1	11/10	5/1	35/14	2/1	0.269
Simplified proteinuria	0(0)	2(10)	1(17)	2(4)	0(0)	
Hematuria+proteinuria	1(100)	15(71)	3(50)	33(67)	1(33)	
Acute nephritis	0(0)	2(10)	1(17)	8(16)	0(0)	
Nephrotic syndrome	0(0)	2(10)	1(17)	6(12)	2(67)	
24hUPro(mg/kg.d^−1^)	25.68	43.17 ± 35.98	55.22 ± 23.67	52.01 ± 43.80	53.46 ± 25.32	0.876
URBC(/HP)	12.00	77.35 ± 140.05	192.42 ± 298.38	130.77 ± 304.42	8.17 ± 12.67	0.783

### Changes in inflammatory indicators and oxidative stress indicators in healthy control group and each group with HSP

The blood inflammatory mediators (IL-6 and TNF-α), lipid peroxidation products (MDA), and antioxidant enzyme systems (SOD and T-AOC) showed statistically significant differences in each group (*P* = 0.000). The levels of IL-6, TNF-α and MDA in the acute phase of HSP children in each group were significantly higher than those in the healthy group, and the levels of SOD and T-AOC were significantly lower than those in the healthy group (*P* = 0.000). Compared with the HSP without GI and renal involvement group, HSP with GI involvement group and healthy group, the levels of IL-6, TNF-α and MDA in the HSPN group were significantly higher(*P* = 0.000), while the levels of SOD and T-AOC were significantly lower (*P* = 0.000). Compared with the HSP without GI and renal involvement group and healthy group, the levels of IL-6, TNF-α and MDA in the HSP with GI involvement group were higher, while the levels of SOD and T-AOC were significantly lower (*P* = 0.000). Compared with the healthy group, the levels of IL-6, TNF-α and MDA in the HSP without GI and renal involvement group were higher, and the levels of SOD and T-AOC were significantly lower (*P* = 0.000), (Table 3).

**Table 3:** Changes of blood inflammatory indicators and oxidative stress indicators in each HSP group and healthy control group (^−^*x ± s*)

	HSP-GI and renal involvement(*n* = 60)	HSP + GI involvement(*n* = 60)	HSPN(*n* = 80)	Healthy group(*n* = 30)	*P* value
IL-6(pg/mL)	10.96 ± 1.47^a^	18.37 ± 1.84^ab^	22.94 ± 3.43^ac^	2.31 ± 0.56	0.000
TNF-α(pg/mL)	11.10 ± 1.31^a^	17.75 ± 2.35 ^ab^	22.26 ± 3.35 ^ac^	3.56 ± 1.25	0.000
MDA(nmol/mL)	2.04 ± 0.12 ^a^	2.96 ± 0.18 ^ab^	3.33 ± 0.42 ^ac^	1.39 ± 0.24	0.000
SOD(U/mL)	44.44 ± 1.93 ^a^	38.64 ± 2.14 ^ab^	34.12 ± 2.73 ^ac^	56.84 ± 4.62	0.000
T-AOC(U/mL)	8.62 ± 0.73 ^a^	6.24 ± 0.63 ^ab^	5.31 ± 0.72 ^ac^	13.68 ± 1.68	0.000

Note: a indicates comparison with the healthy control group, *P* = 0.00

b indicates comparison with the HSP without GI and renal involvement group, *P* = 0.000

c indicates comparison with the HSP without GI and renal involvement group and HSP with GI involvement group, *P* = 0.000

### Correlation analysis of blood IL-6, TNF-α, MDA levels and renal pathological grade in HSPN group

**Fig. 1:**
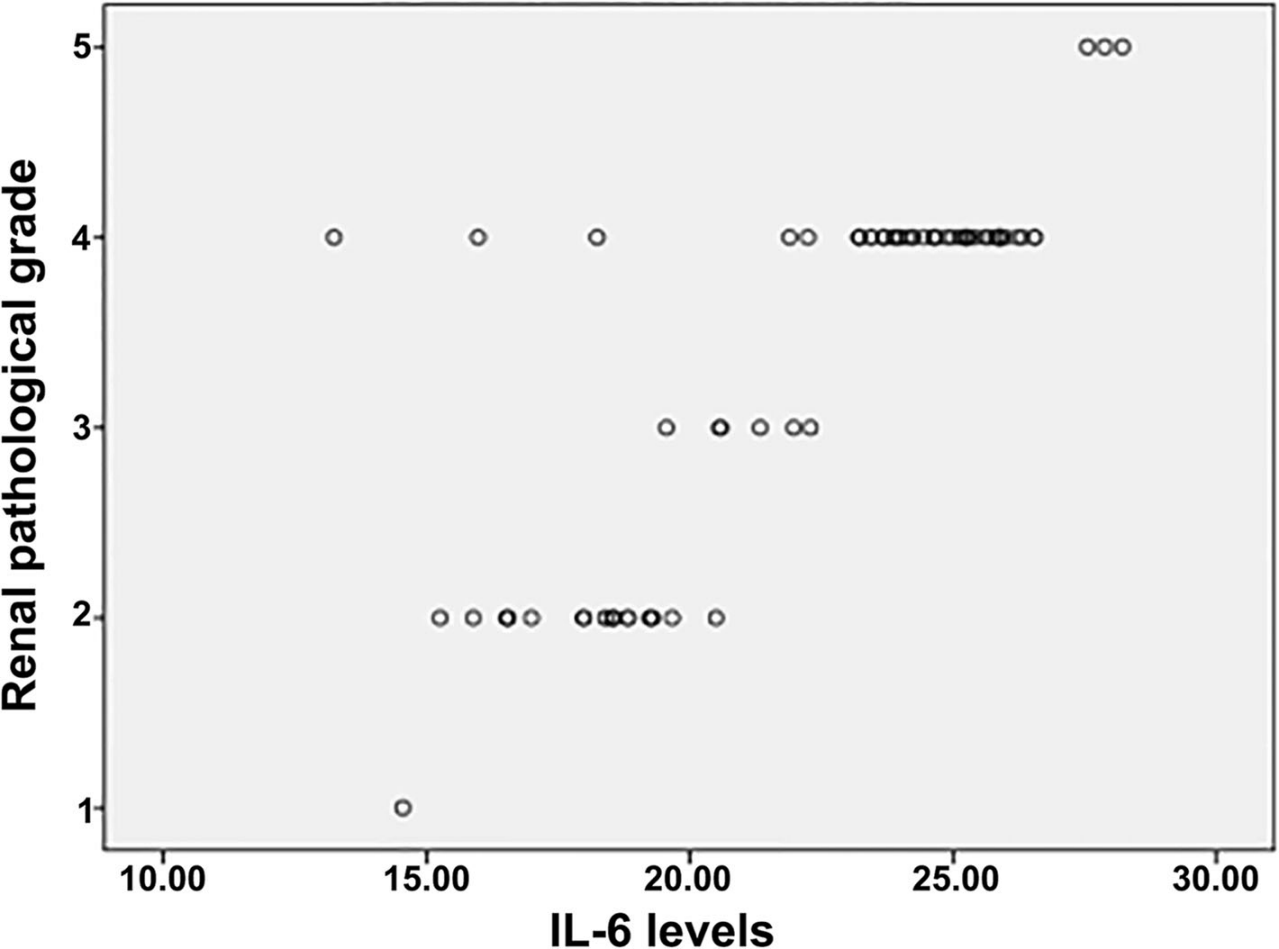
Relationship between levels of serum IL-6 and renal pathological grade in children with HSPN

**Fig. 2:**
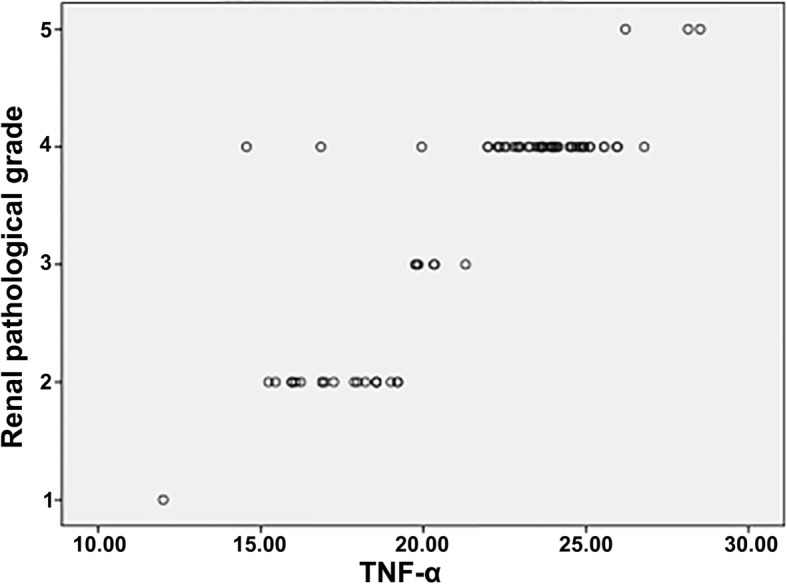
Relationship between levels of serum TNF-α and renal pathological grade in children with HSPN

**Fig. 3:**
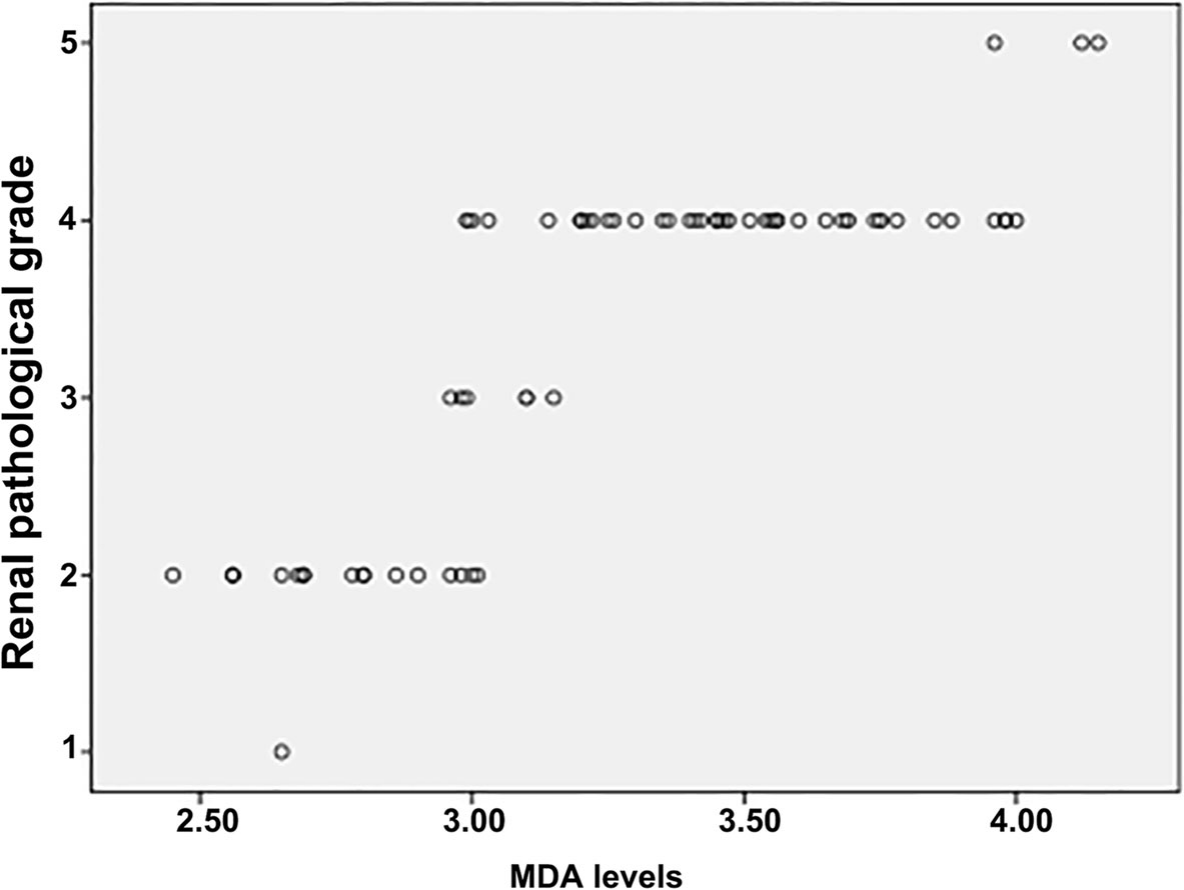
Relationship between levels of plasma MDA and renal pathological grade in children with HSPN

The levels of IL-6, TNF-α and MDA in the HSPN group of patients were positively correlated with the degree of pathological grade (*r* = 0.843, 0.875, 0.070, *P* = 0.000, 0.000, 0.003), see Figs. 1, 2, 3.

### Demographics, clinical parameters in the conventional group and HP group of HSP with GI involvement

No significant difference in age, gender and the severity of disease was observed between the conventional treatment group and the HP group of the HSP with GI involvement (Table 4).

**Table 4:** Comparisons of clinical parameters, glucocorticoid dosage, costs, and renal damage and recurrence rate between the two groups [cases(%),^−^*x ± s*]

	Conventional group(*n* = 30)	HP group(*n* = 30)	*P* value
Age(years)	7.40 ± 2.97	7.77 ± 3.40	0.658
Gender(M/F)	17/13	20/10	0.596
GI bleeding	25(83)	25(83)	1.000
Persistent abdominal pain	5(17)	5(17)	1.000
intussusception	1(3)	3(10)	0.612
Intestinal obstruction	0(0)	1(3)	1.000
Intestinal perforation	1(3)	1(3)	1.000
Acute pancreatitis	1(3)	1(3)	1.000
Arthralgia	13(43)	11(37)	0.792
WBC(×10^9^/L)	14.75 ± 6.12	14.66 ± 6.03	0.953
CRP(mg/L)	9.16 ± 9.44	12.93 ± 16.90	0.292
ALB (g/L)	40.46 ± 6.40	38.86 ± 6.75	0.351
Scr(μmol/L)	27.49 ± 8.68	31.02 ± 10.35	0.157
IgA(g/L)	1.84 ± 0.68	2.02 ± 0.62	0.286
C3(g/L)	1.08 ± 0.29	1.04 ± 0.23	0.496
Fib(g/L)	3.25 ± 2.69	2.46 ± 0.64	0.125
GI syndromes disappeared	13(43)	22(73)	0.035
Time for rash disappearance(d)	8.10 ± 3.54	6.57 ± 2.18	0.048
Time for digestive tract symptom disappearance(d)	9.00 ± 4.35	6.33 ± 4.51	0.023
Daily glucocorticoid dosage (mg/kg)	10.19 ± 5.19	4.74 ± 1.44	0.000
Hospital Costs(thousand yuan)	17.23 ± 8.60	28.40 ± 11.98	0.000
Length of stay (d)	16.5 ± 6.5	17.9 ± 6.5	0.435
Hematuria and/or proteinuria	23(77)	11(37)	0.004
Recurrence of rah	11(37)	6(20)	0.252
Recurrence of abdominal pain	6(20)	5(17)	1.000

In the HSP with GI involvement, on day 4 after treatment, the proportion of patients with disappeared GI symptoms in the HP group was higher than that in the conventional group, and the difference was statistically significant (*P* = 0.035). The time for disappearance of rash and GI symptoms in the HP group was less than that in the conventional group (*P* = 0.048, 0.023). Compared with the conventional group, the children in the HP group had lower glucocorticoid dosage and lower incidences of hematuria and/or proteinuria (*P* = 0.000, 0.004), while hospital costs were significantly higher (*P* = 0.000). There were no significant differences in the length of hospital stay, recurrence rates of rash and abdominal pain between the two groups for three mounth (*P* > 0.05) (Table 4).

### Changes in inflammatory indicators and oxidative stress indicators before and after treatment in the conventional group and HP group of HSP with GI involvement

In the HSP with GI involvement, the levels of IL-6, TNF-α, MDA, SOD and T-AOC in the conventional group and the HP group showed no significant differences before treatment (d0) (*P* > 0.05). After treatment (d4), the levels of IL-6, TNF-α and MDA in the conventional group and the HP group of patients were lower than those before treatment. The SOD and T-AOC levels were recovered and increased compared with those before treatment, but the differences were still statistically significant compared with those in the healthy group (*P* = 0.000). After treatment (d4), the levels of IL-6, TNF-α and MDA in the HP group were lower than those in the conventional group, while the levels of SOD and T-AOC were higher than those in the conventional group (*P* = 0.000) (Table 5).

**Table 5:** Changes of blood inflammatory indicators and oxidative stress indicators before and after treatment in the conventional group and HP group (^−^*x ± s*)

	Conventional group(30)		HP group(*n* = 30)		*P* value
	d0	d4	d0	d4	
IL-6(pg/mL)	18.23 ± 1.99	10.19 ± 1.56^a^	18.45 ± 1.62	7.60 ± 1.15^bc^	0.000
TNF-α(pg/mL)	17.24 ± 2.08	10.32 ± 1.35^a^	17.60 ± 2.77	9.48 ± 0.97^bc^	0.008
MDA(nmol/mL)	2.95 ± 0.19	2.26 ± 0.16^a^	2.97 ± 0.19	2.15 ± 0.18^bc^	0.015
SOD(U/mL)	38.44 ± 2.05	42.71 ± 1.98^a^	38.71 ± 2.26	45.74 ± 2.47^bc^	0.000
T-AOC(U/mL)	6.24 ± 0.54	8.51 ± 0.80^a^	6.20 ± 0.72	9.42 ± 0.71^bc^	0.000

Note: a indicates comparison with the conventional group (d0), *P* = 0.000. b indicates comparison with HP group (d0), *P* = 0.000. c indicates comparison with the conventional group (d4), *P* < 0.047

### Complications of HP and high dose methylprednisolone treatment

There were 30 children treated with HP for a total of 90 times. During HP treatment, there were 3 cases of mild increase in blood pressure and 1 case of hypothermia. The patients with elevated blood pressure were self-relief soon by dynamic monitoring, and the patient with hypothermia got better after keeping warm. No complications such as hypotension, arrhythmia, bleeding, coagulation, thrombocytopenia, catheter related infection occurred. There were 30 children treated with high dose methylprednisolone for a total of 90 times. During the treatment, there were 12 cases of high intraocular pressure, 6 cases of hypertension, and 2 cases of tachycardia.

## Discussion

This study was to investigate the changes of inflammatory and oxidative stress responses in the acute phase of HSP children, the possible protection mechanism of HP for severe HSP with GI and renal involvement. The current research results show that IL-6, TNF-α and MDA might be involved in the acute phase of HSP. The intensity of inflammatory response and oxidative stress were closely related to the degree of illness in HSP. The curative effect of HP during the acute phase was better than that of medical treatment alone, and it can reduce glucocorticoid dosage and prevent renal involvement in severe HSP with GI involvement. The mechanism might be related to the fact that HP can quickly remove IL-6, TNF-α, MDA in HSP children.

The onset of HSP was associated with an imbalance of Th1/Th2 and Th2 preponderance activation, leading to increased secretion of Th2 cytokines. IL-6 is a core factor secreted by Th2 cells, and it can be used as an indicator of HSP activity. IL-6 can stimulate the adhesion, proliferation and differentiation of glomerular mesangial cells of under synergistic action of other cytokines, which in turn lead to glomerular sclerosis [29, 30]. TNF-α is a cytokine that is produced by mononuclear macrophages, and can stimulate immune-active cells to produce inflammatory factors such as interleukins. It can also cause structural changes in vascular endothelial cells, inhibit endothelial cell growth, and change the renal blood flow, leading to glomerular damage. Previous studies showed that the serum TNF-α levels were more significantly increased in children with HSPN, suggesting that TNF-α is closely related to renal injury in HSP [31, 32]. In this study, we found that children with HSP have excessive inflammatory response in vivo, and are closely related to the severity of the disease, the most obvious in HSPN, followed by severe HSP with GI involvement, the HSP without GI and renal involvement is the lightest.

After the damage of vascular endothelial cells, the neutrophils were activated, producing a large number of reactive oxygen species (ROS), further accelerating the progression of HSP [33]. The antioxidant enzyme systems such as T-AOC, SOD and others in the body maintain a dynamic balance of ROS production and clearance. When this balance was broken, lipid peroxidation occurs. MDA is a lipid peroxide that is produced by oxygen free radicals attacking the cell membranes. The levels of MDA were used as one of the indicators of lipid peroxidation degree, which indirectly reflects the degree of endothelial cell damage. Zhong Yanlan [20] reported that serum superoxide anion and MDA levels were significantly increased in children with severe HSP. Li Juan et al. [34] showed that MDA was correlated with the occurrence of HSPN. This study found that compared with the healthy group, the MDA in the acute phase of HSP children in each group were significantly increased, while the SOD and T-AOC were significantly decreased. In addition, the changes in the HSPN group were more obvious than those in the HSP with GI involvement group and the HSP without GI and renal involvement group, and the changes in the HSP with GI involvement group were more obvious than those in the HSP without GI and renal involvement group. This indicated that the intensity of lipid peroxidation in children with HSP was related to the clinical manifestations and severity of the illness.

Oxidative stress and inflammatory response play important roles in the mechanisms of renal damage and proteinuria [35, 36]. Whether the kidney was affected or not and the extent of damage were considered as the most critical factors affecting the long-term prognosis of HSP. Further pathological biopsy of renal tissues helped to determine the degree of renal damage. HSPN was divided into VI according to the International Pediatric Kidney Disease Research Group [7, 27]. In the study, the indication of renal biopsy is more than moderate proteinuria with or without hematuria. Renal biopsies were performed in 80 patients, including 5 cases with simplified proteinuria, 53 cases with proteinuria and hematuria, 11 cases with acute nephritis and 11 cases with nephrotic syndrome. Renal pathology showed 1 case in grade I, 21 cases in grade IIa, 6 cases in grade IIb, 49 cases in grade IIIa, and 3 cases in grade IIIb. There were not statistically different in different pathological grades in 24 h urinary protein excretion and urinary red blood cell counts. This study found that the levels of IL-6, TNF-α, and MDA in the HSPN group were positively correlated with the pathological grade. It was suggested that the inflammatory response and the oxidative stress might be involved in the pathological processes of renal damage during the acute phase of HSP. Meanwhile, the inflammatory response and the oxidative stress may aggravate the clinical progression of children. However, our study mainly focused on HSPN of class IIa and IIIa, and the highest class was IIIb. Changes in inflammatory response and the oxidative stress at higher pathological classes need to be further studied.

Glucocorticoids were mainly used for the treatment of HSP with GI involvement, but it was difficult to be cured in some cases as the inflammatory mediators and oxidative stress products cannot be eliminated in time [37]. Refractory or recurrent HSP has achieved a certain effect on the addition of immunosuppressive agents to glucocorticoid [38, 39]. However, these medicines have a slow onset and are limited in cases of severe acute attacks. HP is a new blood purification method developed in recent years. It used extracorporeal circulation to introduce the patient’s blood into a perfusion device that contains a solid adsorbent to remove exogenous or endogenous toxic substances or morbid substance in the blood by adsorption. Then, the purified blood was returned to the body of the patient [40]. The HA-280 resin hemoperfusion device used a neutral macroporous adsorption resin as an adsorbent. It can effectively remove the inflammatory mediators, antibodies and circulating immune complexes, regulate and reconstruct the immune balance [37]. In this study, there were 50 cases of GI bleeding and 10 cases of persistent abdominal pain in HSP with GI involvement group, including 4 cases of intussusception, 2 cases of intestinal perforation, 2 cases of acute pancreatitis and 1 case of intestinal obstruction. The abdominal pain, digestive tract bleeding, rash and others were all relieved after treatment in both conventional group and HP group of the abdominal HSP, but the symptoms were relieved rapidly in the HP group. In children of the HP group, the glucocorticoid dosage was lower, and the IL-6 and TNF-α, and MDA were decreased more significantly, and the SOD and T-AOC was recovered and increased more significantly. The results suggested that the combination of medicines and HP in HSP with GI involvement improved the sensitivity of the body to medicines and reduced the dosage of glucocorticoids. The GI bleeding in children with HSP was one of the independent risk factors for HSPN [25]. Glucocorticoids can shorten the course of abdominal pain and GI bleeding in HSP, but cannot prevent the kidney involvement [28]. The results of this study suggested that the incidence of hematuria and/or proteinuria within 3 months of the disease was lower in the HP group, and the mechanisms might be related that by rapid elimination of IL-6, TNF-α and MDA by HP, preventing the damage of inflammatory mediators and lipid oxidative products on glomerular endothelial cells and improving the microcirculation of renal tissues to protect the renal function.

## Conclusions

In conclusion, HP can reduce glucocorticoid dosage and the rate of renal involvement in children with severe HSP with GI involvement. The mechanism may be related to the fact that HP can effectively remove IL-6, TNF-α, MDA in HSP children. But the treatment of HP is costly and invasive, limiting clinical use. However, HP treatment has no obvious complications, which is one of the safe and effective methods for the treatment of severe HSP with GI involvement, especially those with GI complications, high risk of kidney involvement and restriction of glucocorticoid usage during the acute phase. Nevertheless, its long-term efficacy should be further clarified in larger sample size by prospective randomized controlled clinical trials.

## Data Availability

The datasets used and analysed during the current study are available from the corresponding author on reasonable request.

## Abbreviations

HP: Hemoperfusion
HSP: Henoch-Schönlein purpura
HSPN: Henoch-Schönlein purpura nephritis
MDA: Malondialdehyde
ROS: Reactive oxygen species
SOD: Superoxide dismutase
T-AOC: Total anti-oxidant capability
